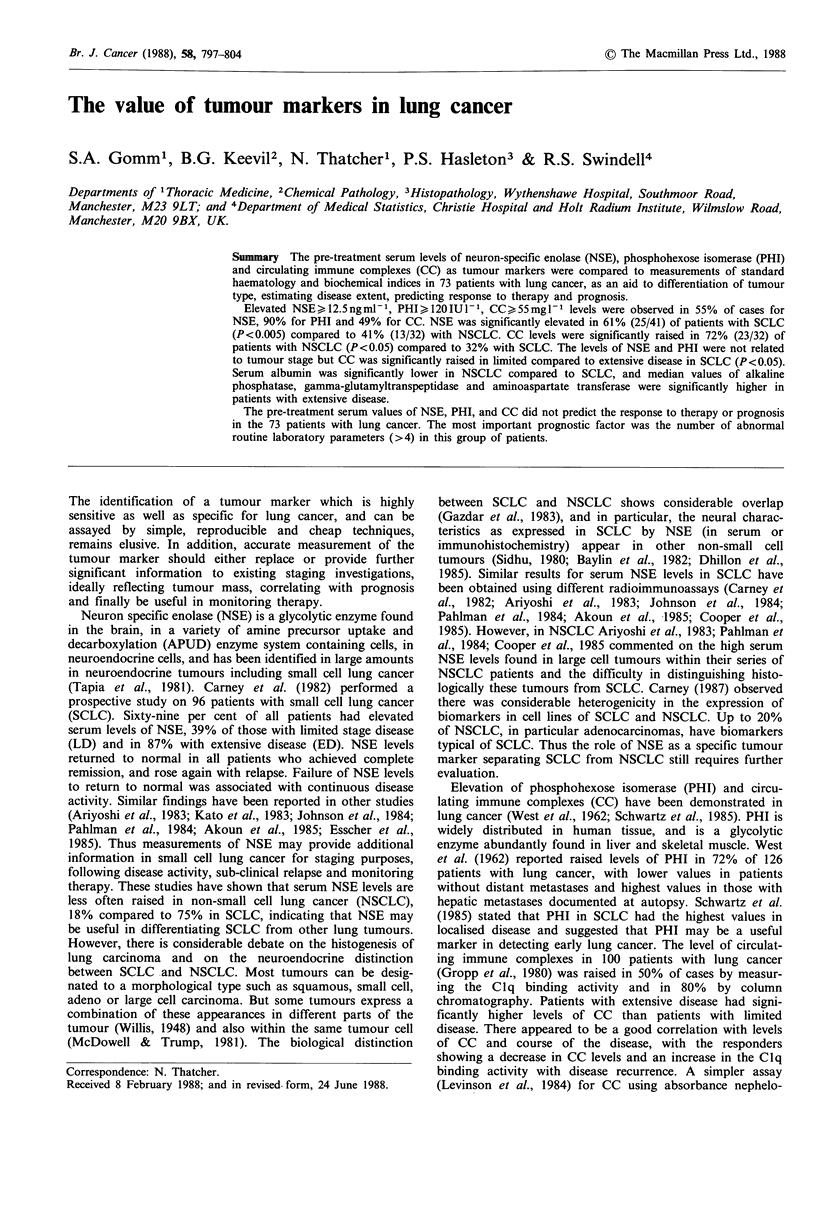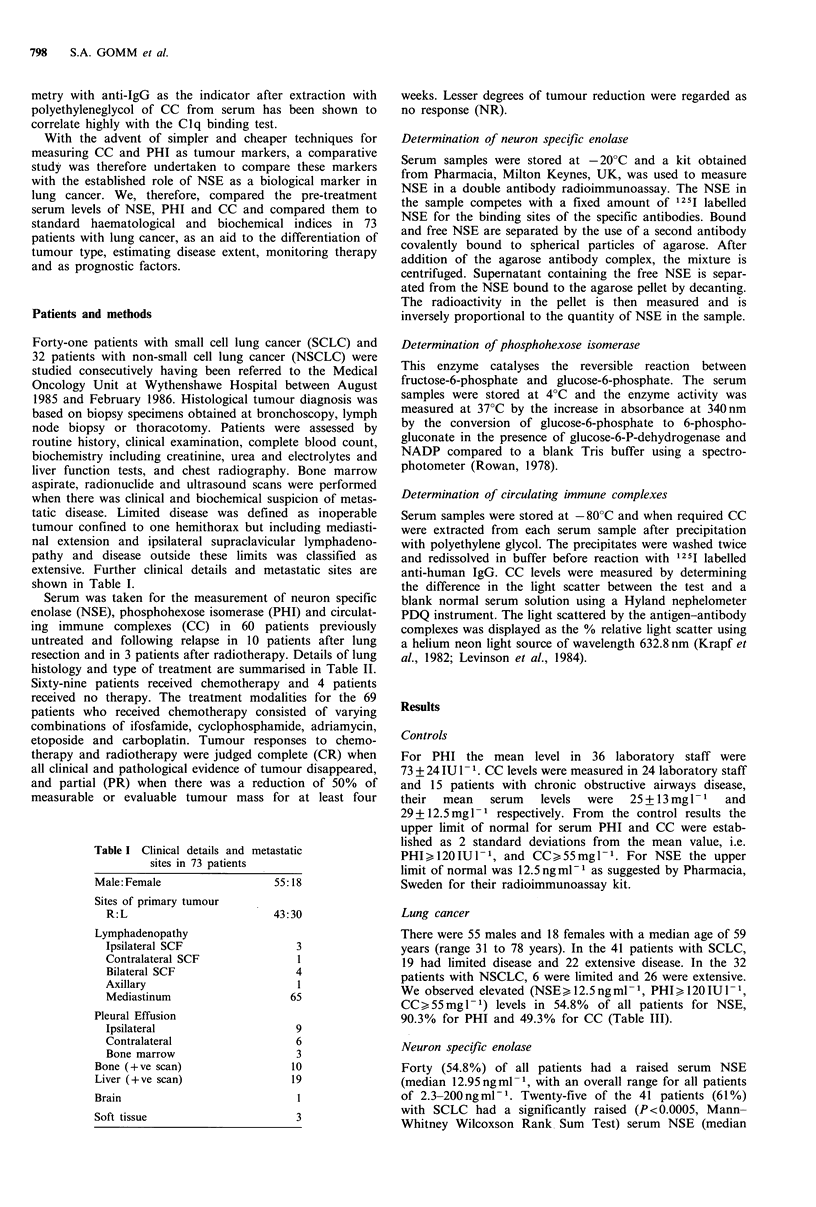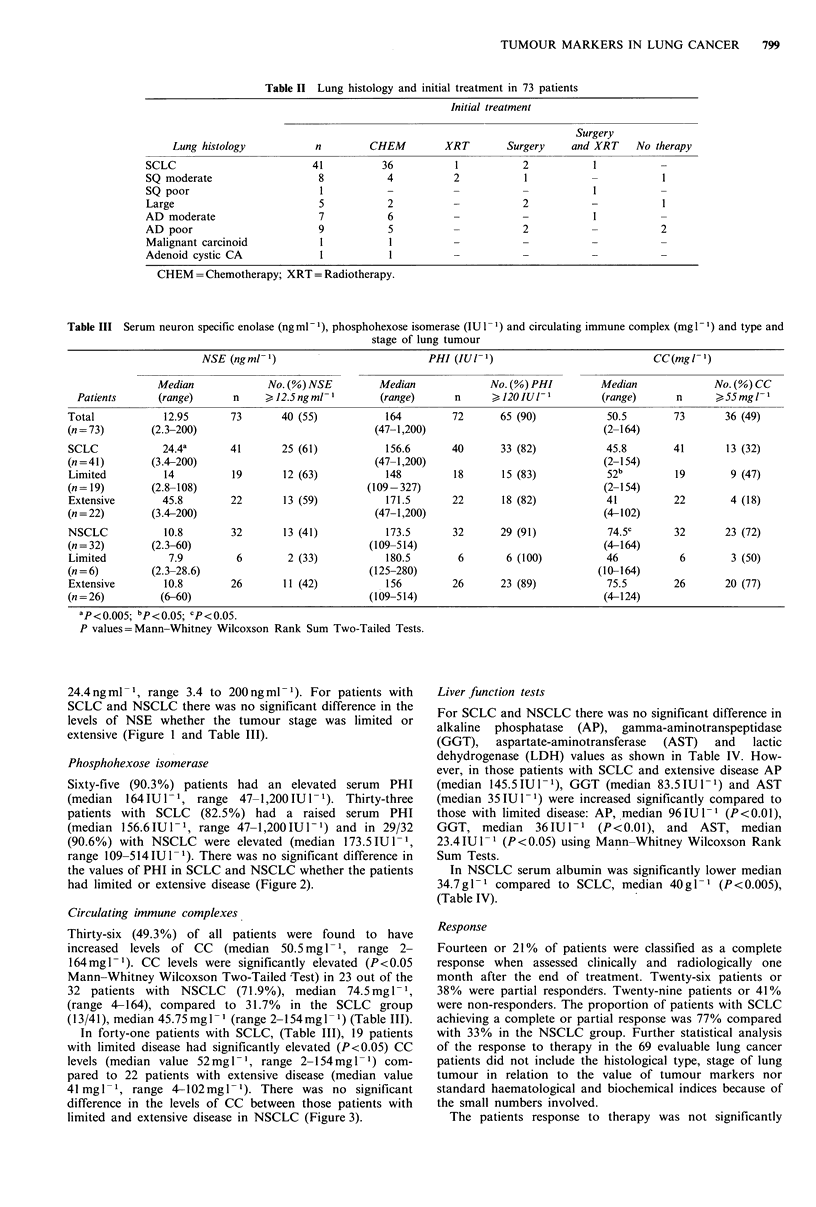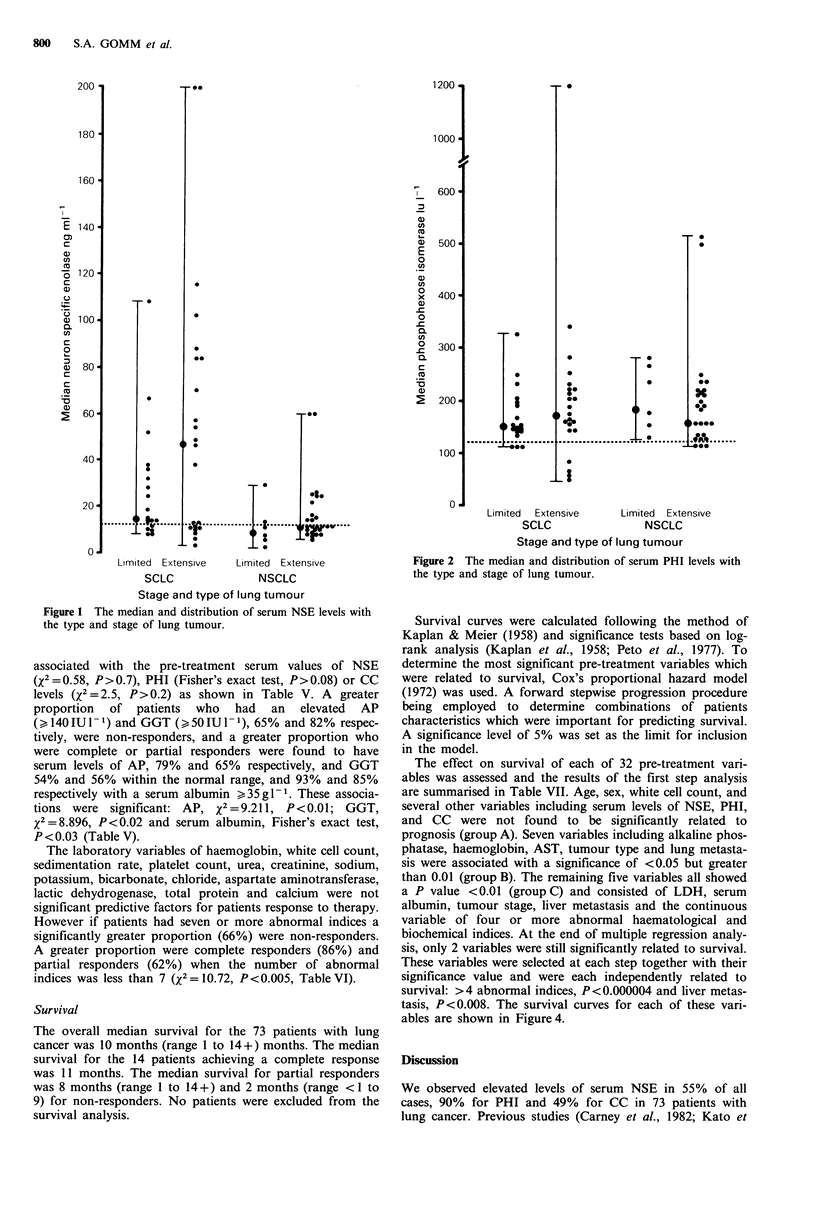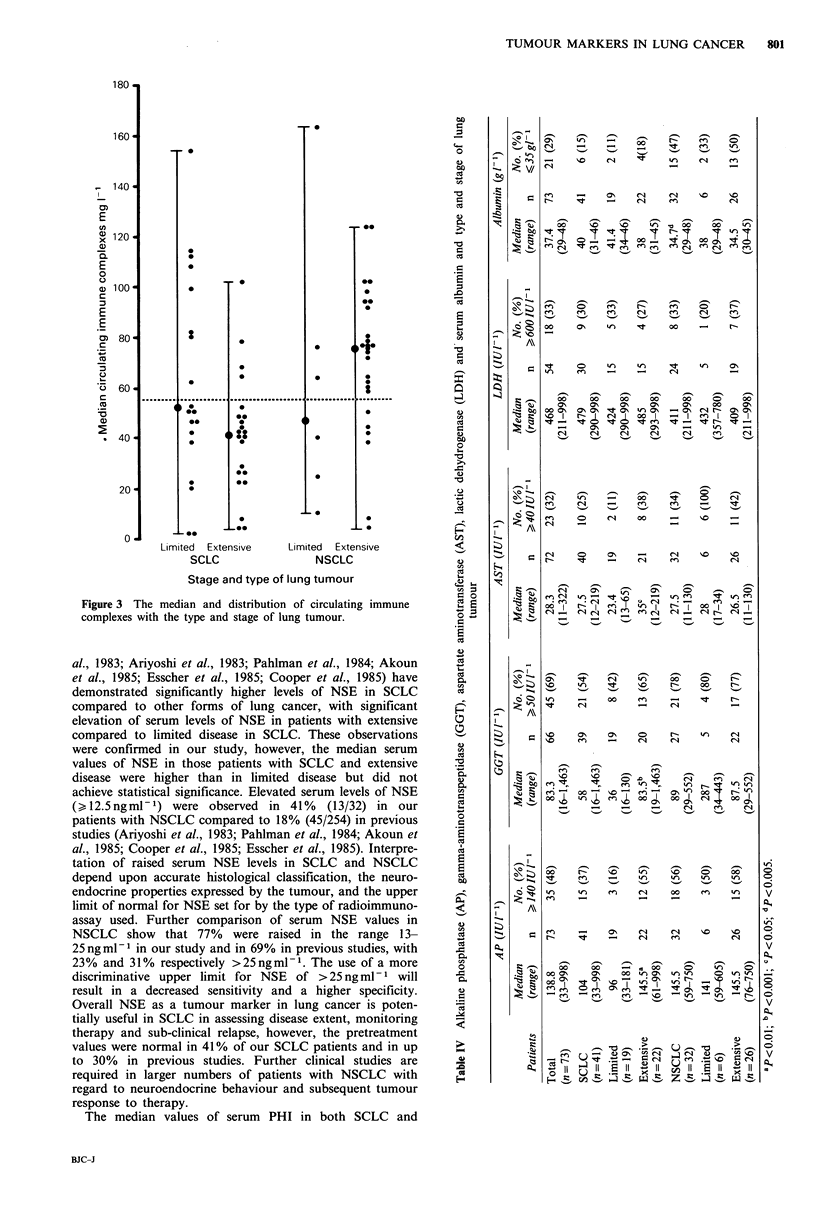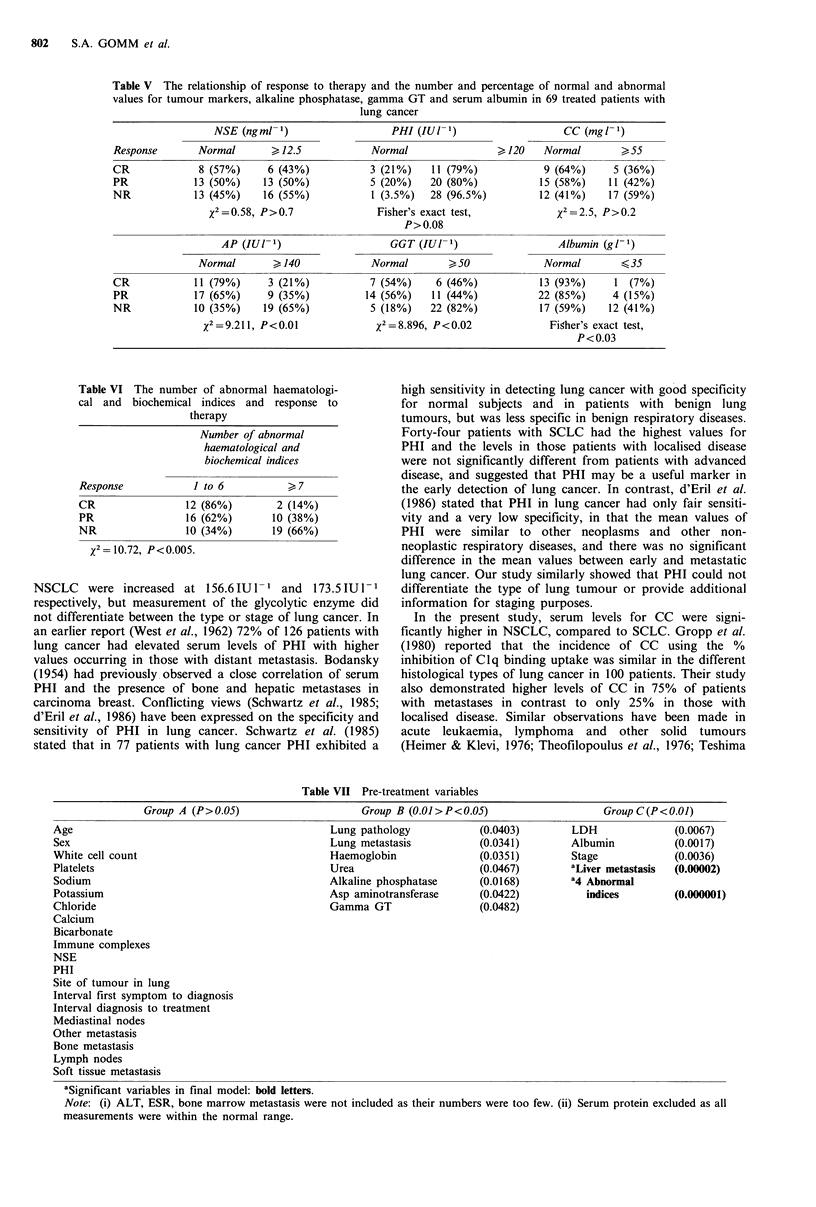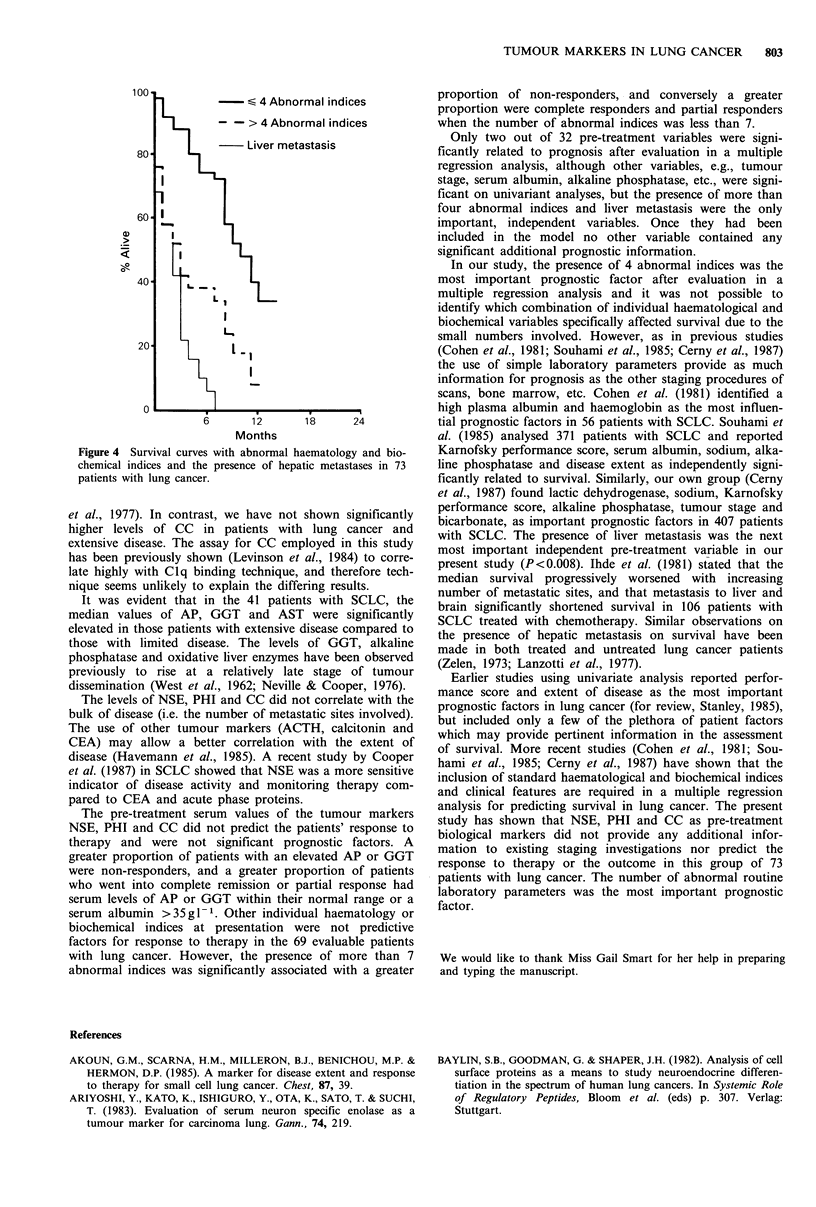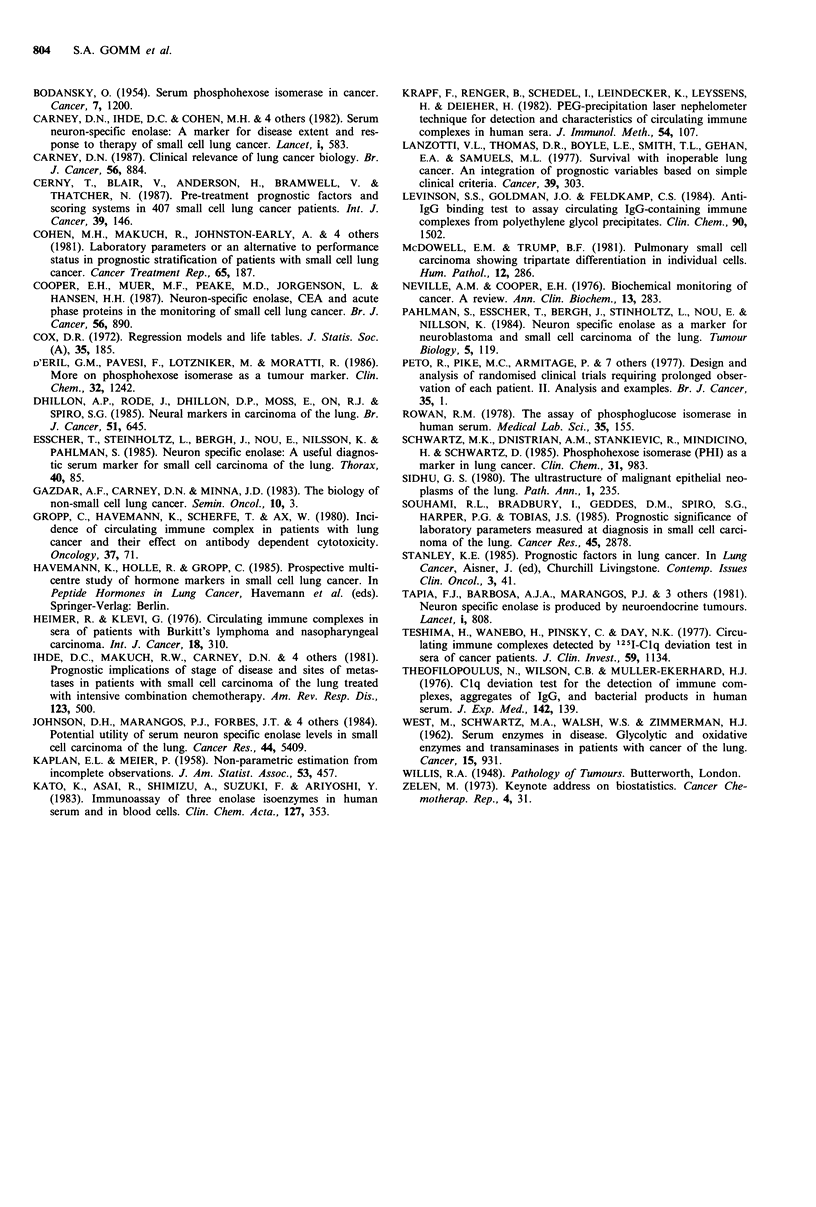# The value of tumour markers in lung cancer.

**DOI:** 10.1038/bjc.1988.312

**Published:** 1988-12

**Authors:** S. A. Gomm, B. G. Keevil, N. Thatcher, P. S. Hasleton, R. S. Swindell

**Affiliations:** Department of Thoracic Medicine, Wynthenshawe Hospital, Manchester, UK.

## Abstract

The pre-treatment serum levels of neuron-specific enolase (NSE), phosphohexose isomerase (PHI) and circulating immune complexes (CC) as tumour markers were compared to measurements of standard haematology and biochemical indices in 73 patients with lung cancer, as an aid to differentiation of tumour type, estimating disease extent, predicting response to therapy and prognosis. Elevated NSE greater than or equal to 12.5 ng ml-1, PHI greater than or equal to 55 mgl-1 levels were observed in 55% of cases for NSE, 90% for PHI and 49% for CC. NSE was significantly elevated in 61% (25/41) of patients with SCLC (P less than 0.005) compared to 41% (13/32) with NSCLC. CC levels were significantly raised in 72% (23/32) of patients with NSCLC (P less than 0.05) compared to 32% with SCLC. The levels of NSE and PHI were not related to tumour stage but CC was significantly raised in limited compared to extensive disease in SCLC (P less than 0.05). Serum albumin was significantly lower in NSCLC compared to SCLC, and median values of alkaline phosphatase, gamma-glutamyltranspeptidase and aminoaspartate transferase were significantly higher in patients with extensive disease. The pre-treatment serum values of NSE, PHI, and CC did not predict the response to therapy or prognosis in the 73 patients with lung cancer. The most important prognostic factor was the number of abnormal routine laboratory parameters (greater than 4) in this group of patients.


Br. J. Cancer (1988), 58, 797–804 © The Macmillan Press Ltd., 1988

### The value of tumour markers in lung cancer

S.A. Gomm[1], B.G. Keevil[2], N. Thatcher[1], P.S. Hasleton[3] & R.S. Swindell[4]

*Departments of [1]Thoracic Medicine, [2]Chemical Pathology, [3]Histopathology, Wythenshawe Hospital, Southmoor Road, Manchester, M23 9LT; and [4]Department of Medical Statistics, Christie Hospital and Holt Radium Institute, Wilmslow Road, Manchester, M20 9BX, UK.*

**Summary** The pre-treatment serum levels of neuron-specific enolase (NSE), phosphohexose isomerase (PHI) and circulating immune complexes (CC) as tumour markers were compared to measurements of standard haematology and biochemical indices in 73 patients with lung cancer, as an aid to differentiation of tumour type, estimating disease extent, predicting response to therapy and prognosis.

Elevated NSE$\geqslant$12.5 ng ml$^{-1}$, PHI$\geqslant$120 IU l$^{-1}$, CC$\geqslant$55 mg l$^{-1}$ levels were observed in 55% of cases for NSE, 90% for PHI and 49% for CC. NSE was significantly elevated in 61% (25/41) of patients with SCLC ($P<0.005$) compared to 41% (13/32) with NSCLC. CC levels were significantly raised in 72% (23/32) of patients with NSCLC ($P<0.05$) compared to 32% with SCLC. The levels of NSE and PHI were not related to tumour stage but CC was significantly raised in limited compared to extensive disease in SCLC ($P<0.05$). Serum albumin was significantly lower in NSCLC compared to SCLC, and median values of alkaline phosphatase, gamma-glutamyltranspeptidase and aminoaspartate transferase were significantly higher in patients with extensive disease.

The pre-treatment serum values of NSE, PHI, and CC did not predict the response to therapy or prognosis in the 73 patients with lung cancer. The most important prognostic factor was the number of abnormal routine laboratory parameters (>4) in this group of patients.

The identification of a tumour marker which is highly sensitive as well as specific for lung cancer, and can be assayed by simple, reproducible and cheap techniques, remains elusive. In addition, accurate measurement of the tumour marker should either replace or provide further significant information to existing staging investigations, ideally reflecting tumour mass, correlating with prognosis and finally be useful in monitoring therapy.

Neuron specific enolase (NSE) is a glycolytic enzyme found in the brain, in a variety of amine precursor uptake and decarboxylation (APUD) enzyme system containing cells, in neuroendocrine cells, and has been identified in large amounts in neuroendocrine tumours including small cell lung cancer (Tapia *et al.*, 1981). Carney *et al.* (1982) performed a prospective study on 96 patients with small cell lung cancer (SCLC). Sixty-nine per cent of all patients had elevated serum levels of NSE, 39% of those with limited stage disease (LD) and in 87% with extensive disease (ED). NSE levels returned to normal in all patients who achieved complete remission, and rose again with relapse. Failure of NSE levels to return to normal was associated with continuous disease activity. Similar findings have been reported in other studies (Ariyoshi *et al.*, 1983; Kato *et al.*, 1983; Johnson *et al.*, 1984; Pahlman *et al.*, 1984; Akoun *et al.*, 1985; Esscher *et al.*, 1985). Thus measurements of NSE may provide additional information in small cell lung cancer for staging purposes, following disease activity, sub-clinical relapse and monitoring therapy. These studies have shown that serum NSE levels are less often raised in non-small cell lung cancer (NSCLC), 18% compared to 75% in SCLC, indicating that NSE may be useful in differentiating SCLC from other lung tumours. However, there is considerable debate on the histogenesis of lung carcinoma and on the neuroendocrine distinction between SCLC and NSCLC. Most tumours can be designated to a morphological type such as squamous, small cell, adeno or large cell carcinoma. But some tumours express a combination of these appearances in different parts of the tumour (Willis, 1948) and also within the same tumour cell (McDowell & Trump, 1981). The biological distinction between SCLC and NSCLC shows considerable overlap (Gazdar *et al.*, 1983), and in particular, the neural characteristics as expressed in SCLC by NSE (in serum or immunohistochemistry) appear in other non-small cell tumours (Sidhu, 1980; Baylin *et al.*, 1982; Dhillon *et al.*, 1985). Similar results for serum NSE levels in SCLC have been obtained using different radioimmunoassays (Carney *et al.*, 1982; Ariyoshi *et al.*, 1983; Johnson *et al.*, 1984; Pahlman *et al.*, 1984; Akoun *et al.*, 1985; Cooper *et al.*, 1985). However, in NSCLC Ariyoshi *et al.*, 1983; Pahlman *et al.*, 1984; Cooper *et al.*, 1985 commented on the high serum NSE levels found in large cell tumours within their series of NSCLC patients and the difficulty in distinguishing histologically these tumours from SCLC. Carney (1987) observed there was considerable heterogenicity in the expression of biomarkers in cell lines of SCLC and NSCLC. Up to 20% of NSCLC, in particular adenocarcinomas, have biomarkers typical of SCLC. Thus the role of NSE as a specific tumour marker separating SCLC from NSCLC still requires further evaluation.

Elevation of phosphohexose isomerase (PHI) and circulating immune complexes (CC) have been demonstrated in lung cancer (West *et al.*, 1962; Schwartz *et al.*, 1985). PHI is widely distributed in human tissue, and is a glycolytic enzyme abundantly found in liver and skeletal muscle. West *et al.* (1962) reported raised levels of PHI in 72% of 126 patients with lung cancer, with lower values in patients without distant metastases and highest values in those with hepatic metastases documented at autopsy. Schwartz *et al.* (1985) stated that PHI in SCLC had the highest values in localised disease and suggested that PHI may be a useful marker in detecting early lung cancer. The level of circulating immune complexes in 100 patients with lung cancer (Gropp *et al.*, 1980) was raised in 50% of cases by measuring the C1q binding activity and in 80% by column chromatography. Patients with extensive disease had significantly higher levels of CC than patients with limited disease. There appeared to be a good correlation with levels of CC and course of the disease, with the responders showing a decrease in CC levels and an increase in the C1q binding activity with disease recurrence. A simpler assay (Levinson *et al.*, 1984) for CC using absorbance nephelo-

Correspondence: N. Thatcher.
Received 8 February 1988; and in revised form, 24 June 1988.

metry with anti-IgG as the indicator after extraction with polyethyleneglycol of CC from serum has been shown to correlate highly with the C1q binding test.

With the advent of simpler and cheaper techniques for measuring CC and PHI as tumour markers, a comparative study was therefore undertaken to compare these markers with the established role of NSE as a biological marker in lung cancer. We, therefore, compared the pre-treatment serum levels of NSE, PHI and CC and compared them to standard haematological and biochemical indices in 73 patients with lung cancer, as an aid to the differentiation of tumour type, estimating disease extent, monitoring therapy and as prognostic factors.

### Patients and methods

Forty-one patients with small cell lung cancer (SCLC) and 32 patients with non-small cell lung cancer (NSCLC) were studied consecutively having been referred to the Medical Oncology Unit at Wythenshawe Hospital between August 1985 and February 1986. Histological tumour diagnosis was based on biopsy specimens obtained at bronchoscopy, lymph node biopsy or thoracotomy. Patients were assessed by routine history, clinical examination, complete blood count, biochemistry including creatinine, urea and electrolytes and liver function tests, and chest radiography. Bone marrow aspirate, radionuclide and ultrasound scans were performed when there was clinical and biochemical suspicion of metastatic disease. Limited disease was defined as inoperable tumour confined to one hemithorax but including mediastinal extension and ipsilateral supraclavicular lymphadenopathy and disease outside these limits was classified as extensive. Further clinical details and metastatic sites are shown in Table I.

Serum was taken for the measurement of neuron specific enolase (NSE), phosphohexose isomerase (PHI) and circulating immune complexes (CC) in 60 patients previously untreated and following relapse in 10 patients after lung resection and in 3 patients after radiotherapy. Details of lung histology and type of treatment are summarised in Table II. Sixty-nine patients received chemotherapy and 4 patients received no therapy. The treatment modalities for the 69 patients who received chemotherapy consisted of varying combinations of ifosfamide, cyclophosphamide, adriamycin, etoposide and carboplatin. Tumour responses to chemotherapy and radiotherapy were judged complete (CR) when all clinical and pathological evidence of tumour disappeared, and partial (PR) when there was a reduction of 50% of measurable or evaluable tumour mass for at least four weeks. Lesser degrees of tumour reduction were regarded as no response (NR).

**Table I** Clinical details and metastatic sites in 73 patients

| | |
|---|---|
| Male:Female | 55:18 |
| Sites of primary tumour | |
| R:L | 43:30 |
| Lymphadenopathy | |
| Ipsilateral SCF | 3 |
| Contralateral SCF | 1 |
| Bilateral SCF | 4 |
| Axillary | 1 |
| Mediastinum | 65 |
| Pleural Effusion | |
| Ipsilateral | 9 |
| Contralateral | 6 |
| Bone marrow | 3 |
| Bone (+ve scan) | 10 |
| Liver (+ve scan) | 19 |
| Brain | 1 |
| Soft tissue | 3 |

### *Determination of neuron specific enolase*

Serum samples were stored at $-20^{\circ}$C and a kit obtained from Pharmacia, Milton Keynes, UK, was used to measure NSE in a double antibody radioimmunoassay. The NSE in the sample competes with a fixed amount of $^{125}$I labelled NSE for the binding sites of the specific antibodies. Bound and free NSE are separated by the use of a second antibody covalently bound to spherical particles of agarose. After addition of the agarose antibody complex, the mixture is centrifuged. Supernatant containing the free NSE is separated from the NSE bound to the agarose pellet by decanting. The radioactivity in the pellet is then measured and is inversely proportional to the quantity of NSE in the sample.

### *Determination of phosphohexose isomerase*

This enzyme catalyses the reversible reaction between fructose-6-phosphate and glucose-6-phosphate. The serum samples were stored at 4°C and the enzyme activity was measured at 37°C by the increase in absorbance at 340 nm by the conversion of glucose-6-phosphate to 6-phosphogluconate in the presence of glucose-6-P-dehydrogenase and NADP compared to a blank Tris buffer using a spectrophotometer (Rowan, 1978).

### *Determination of circulating immune complexes*

Serum samples were stored at $-80^{\circ}$C and when required CC were extracted from each serum sample after precipitation with polyethylene glycol. The precipitates were washed twice and redissolved in buffer before reaction with $^{125}$I labelled anti-human IgG. CC levels were measured by determining the difference in the light scatter between the test and a blank normal serum solution using a Hyland nephelometer PDQ instrument. The light scattered by the antigen–antibody complexes was displayed as the % relative light scatter using a helium neon light source of wavelength 632.8 nm (Krapf *et al.*, 1982; Levinson *et al.*, 1984).

### Results

### *Controls*

For PHI the mean level in 36 laboratory staff were $73 \pm 24\,IU\,l^{-1}$. CC levels were measured in 24 laboratory staff and 15 patients with chronic obstructive airways disease, their mean serum levels were $25 \pm 13\,mg\,l^{-1}$ and $29 \pm 12.5\,mg\,l^{-1}$ respectively. From the control results the upper limit of normal for serum PHI and CC were established as 2 standard deviations from the mean value, i.e. $PHI \geqslant 120\,IU\,l^{-1}$, and $CC \geqslant 55\,mg\,l^{-1}$. For NSE the upper limit of normal was $12.5\,ng\,ml^{-1}$ as suggested by Pharmacia, Sweden for their radioimmunoassay kit.

### *Lung cancer*

There were 55 males and 18 females with a median age of 59 years (range 31 to 78 years). In the 41 patients with SCLC, 19 had limited disease and 22 extensive disease. In the 32 patients with NSCLC, 6 were limited and 26 were extensive. We observed elevated ($NSE \geqslant 12.5\,ng\,ml^{-1}$, $PHI \geqslant 120\,IU\,l^{-1}$, $CC \geqslant 55\,mg\,l^{-1}$) levels in 54.8% of all patients for NSE, 90.3% for PHI and 49.3% for CC (Table III).

### *Neuron specific enolase*

Forty (54.8%) of all patients had a raised serum NSE (median $12.95\,ng\,ml^{-1}$, with an overall range for all patients of $2.3$–$200\,ng\,ml^{-1}$. Twenty-five of the 41 patients (61%) with SCLC had a significantly raised ($P < 0.0005$, Mann–Whitney Wilcoxson Rank Sum Test) serum NSE (median

**Table II** Lung histology and initial treatment in 73 patients

| *Lung histology* | *n* | *Initial treatment* *CHEM* | *XRT* | *Surgery* | *Surgery and XRT* | *No therapy* |
|---|---|---|---|---|---|---|
| SCLC | 41 | 36 | 1 | 2 | 1 | – |
| SQ moderate | 8 | 4 | 2 | 1 | – | 1 |
| SQ poor | 1 | – | – | – | 1 | – |
| Large | 5 | 2 | – | 2 | – | 1 |
| AD moderate | 7 | 6 | – | – | 1 | – |
| AD poor | 9 | 5 | – | 2 | – | 2 |
| Malignant carcinoid | 1 | 1 | – | – | – | – |
| Adenoid cystic CA | 1 | 1 | – | – | – | – |

CHEM = Chemotherapy; XRT = Radiotherapy.

**Table III** Serum neuron specific enolase ($ng\,ml^{-1}$), phosphohexose isomerase ($IU\,l^{-1}$) and circulating immune complex ($mg\,l^{-1}$) and type and stage of lung tumour

| | *NSE* ($ng\,ml^{-1}$) | | | *PHI* ($IU\,l^{-1}$) | | | *CC* ($mg\,l^{-1}$) | | |
|---|---|---|---|---|---|---|---|---|---|
| *Patients* | *Median (range)* | n | *No. (%) NSE* $\geqslant 12.5\,ng\,ml^{-1}$ | *Median (range)* | n | *No. (%) PHI* $\geqslant 120\,IU\,l^{-1}$ | *Median (range)* | n | *No. (%) CC* $\geqslant 55\,mg\,l^{-1}$ |
| Total ($n$=73) | 12.95 (2.3–200) | 73 | 40 (55) | 164 (47–1,200) | 72 | 65 (90) | 50.5 (2–164) | 73 | 36 (49) |
| SCLC ($n$=41) | 24.4[a] (3.4–200) | 41 | 25 (61) | 156.6 (47–1,200) | 40 | 33 (82) | 45.8 (2–154) | 41 | 13 (32) |
| Limited ($n$=19) | 14 (2.8–108) | 19 | 12 (63) | 148 (109 – 327) | 18 | 15 (83) | 52[b] (2–154) | 19 | 9 (47) |
| Extensive ($n$=22) | 45.8 (3.4–200) | 22 | 13 (59) | 171.5 (47–1,200) | 22 | 18 (82) | 41 (4–102) | 22 | 4 (18) |
| NSCLC ($n$=32) | 10.8 (2.3–60) | 32 | 13 (41) | 173.5 (109–514) | 32 | 29 (91) | 74.5[c] (4–164) | 32 | 23 (72) |
| Limited ($n$=6) | 7.9 (2.3–28.6) | 6 | 2 (33) | 180.5 (125–280) | 6 | 6 (100) | 46 (10–164) | 6 | 3 (50) |
| Extensive ($n$=26) | 10.8 (6–60) | 26 | 11 (42) | 156 (109–514) | 26 | 23 (89) | 75.5 (4–124) | 26 | 20 (77) |

[a]$P<0.005$; [b]$P<0.05$; [c]$P<0.05$.
$P$ values = Mann–Whitney Wilcoxson Rank Sum Two-Tailed Tests.

24.4 $ng\,ml^{-1}$, range 3.4 to 200 $ng\,ml^{-1}$). For patients with SCLC and NSCLC there was no significant difference in the levels of NSE whether the tumour stage was limited or extensive (Figure 1 and Table III).

*Phosphohexose isomerase*

Sixty-five (90.3%) patients had an elevated serum PHI (median 164 $IU\,l^{-1}$, range 47–1,200 $IU\,l^{-1}$). Thirty-three patients with SCLC (82.5%) had a raised serum PHI (median 156.6 $IU\,l^{-1}$, range 47–1,200 $IU\,l^{-1}$) and in 29/32 (90.6%) with NSCLC were elevated (median 173.5 $IU\,l^{-1}$, range 109–514 $IU\,l^{-1}$). There was no significant difference in the values of PHI in SCLC and NSCLC whether the patients had limited or extensive disease (Figure 2).

*Circulating immune complexes*

Thirty-six (49.3%) of all patients were found to have increased levels of CC (median 50.5 $mg\,l^{-1}$, range 2–164 $mg\,l^{-1}$). CC levels were significantly elevated ($P<0.05$ Mann–Whitney Wilcoxson Two-Tailed Test) in 23 out of the 32 patients with NSCLC (71.9%), median 74.5 $mg\,l^{-1}$, (range 4–164), compared to 31.7% in the SCLC group (13/41), median 45.75 $mg\,l^{-1}$ (range 2–154 $mg\,l^{-1}$) (Table III).

In forty-one patients with SCLC, (Table III), 19 patients with limited disease had significantly elevated ($P<0.05$) CC levels (median value 52 $mg\,l^{-1}$, range 2–154 $mg\,l^{-1}$) compared to 22 patients with extensive disease (median value 41 $mg\,l^{-1}$, range 4–102 $mg\,l^{-1}$). There was no significant difference in the levels of CC between those patients with limited and extensive disease in NSCLC (Figure 3).

*Liver function tests*

For SCLC and NSCLC there was no significant difference in alkaline phosphatase (AP), gamma-aminotranspeptidase (GGT), aspartate-aminotransferase (AST) and lactic dehydrogenase (LDH) values as shown in Table IV. However, in those patients with SCLC and extensive disease AP (median 145.5 $IU\,l^{-1}$), GGT (median 83.5 $IU\,l^{-1}$) and AST (median 35 $IU\,l^{-1}$) were increased significantly compared to those with limited disease: AP, median 96 $IU\,l^{-1}$ ($P<0.01$), GGT, median 36 $IU\,l^{-1}$ ($P<0.01$), and AST, median 23.4 $IU\,l^{-1}$ ($P<0.05$) using Mann–Whitney Wilcoxson Rank Sum Tests.

In NSCLC serum albumin was significantly lower median 34.7 $g\,l^{-1}$ compared to SCLC, median 40 $g\,l^{-1}$ ($P<0.005$), (Table IV).

*Response*

Fourteen or 21% of patients were classified as a complete response when assessed clinically and radiologically one month after the end of treatment. Twenty-six patients or 38% were partial responders. Twenty-nine patients or 41% were non-responders. The proportion of patients with SCLC achieving a complete or partial response was 77% compared with 33% in the NSCLC group. Further statistical analysis of the response to therapy in the 69 evaluable lung cancer patients did not include the histological type, stage of lung tumour in relation to the value of tumour markers nor standard haematological and biochemical indices because of the small numbers involved.

The patients response to therapy was not significantly

**Figure 1** The median and distribution of serum NSE levels with the type and stage of lung tumour.

**Figure 2** The median and distribution of serum PHI levels with the type and stage of lung tumour.

associated with the pre-treatment serum values of NSE ($\chi^2=0.58$, $P>0.7$), PHI (Fisher's exact test, $P>0.08$) or CC levels ($\chi^2=2.5$, $P>0.2$) as shown in Table V. A greater proportion of patients who had an elevated AP ($\geqslant 140\,IU\,l^{-1}$) and GGT ($\geqslant 50\,IU\,l^{-1}$), 65% and 82% respectively, were non-responders, and a greater proportion who were complete or partial responders were found to have serum levels of AP, 79% and 65% respectively, and GGT 54% and 56% within the normal range, and 93% and 85% respectively with a serum albumin $\geqslant 35\,g\,l^{-1}$. These associations were significant: AP, $\chi^2=9.211$, $P<0.01$; GGT, $\chi^2=8.896$, $P<0.02$ and serum albumin, Fisher's exact test, $P<0.03$ (Table V).

The laboratory variables of haemoglobin, white cell count, sedimentation rate, platelet count, urea, creatinine, sodium, potassium, bicarbonate, chloride, aspartate aminotransferase, lactic dehydrogenase, total protein and calcium were not significant predictive factors for patients response to therapy. However if patients had seven or more abnormal indices a significantly greater proportion (66%) were non-responders. A greater proportion were complete responders (86%) and partial responders (62%) when the number of abnormal indices was less than 7 ($\chi^2=10.72$, $P<0.005$, Table VI).

### *Survival*

The overall median survival for the 73 patients with lung cancer was 10 months (range 1 to 14+) months. The median survival for the 14 patients achieving a complete response was 11 months. The median survival for partial responders was 8 months (range 1 to 14+) and 2 months (range <1 to 9) for non-responders. No patients were excluded from the survival analysis.

Survival curves were calculated following the method of Kaplan & Meier (1958) and significance tests based on log-rank analysis (Kaplan *et al.*, 1958; Peto *et al.*, 1977). To determine the most significant pre-treatment variables which were related to survival, Cox's proportional hazard model (1972) was used. A forward stepwise progression procedure being employed to determine combinations of patients characteristics which were important for predicting survival. A significance level of 5% was set as the limit for inclusion in the model.

The effect on survival of each of 32 pre-treatment variables was assessed and the results of the first step analysis are summarised in Table VII. Age, sex, white cell count, and several other variables including serum levels of NSE, PHI, and CC were not found to be significantly related to prognosis (group A). Seven variables including alkaline phosphatase, haemoglobin, AST, tumour type and lung metastasis were associated with a significance of <0.05 but greater than 0.01 (group B). The remaining five variables all showed a $P$ value <0.01 (group C) and consisted of LDH, serum albumin, tumour stage, liver metastasis and the continuous variable of four or more abnormal haematological and biochemical indices. At the end of multiple regression analysis, only 2 variables were still significantly related to survival. These variables were selected at each step together with their significance value and were each independently related to survival: >4 abnormal indices, $P<0.000004$ and liver metastasis, $P<0.008$. The survival curves for each of these variables are shown in Figure 4.

### Discussion

We observed elevated levels of serum NSE in 55% of all cases, 90% for PHI and 49% for CC in 73 patients with lung cancer. Previous studies (Carney *et al.*, 1982; Kato *et*

**Figure 3** The median and distribution of circulating immune complexes with the type and stage of lung tumour.

*al.*, 1983; Ariyoshi *et al.*, 1983; Pahlman *et al.*, 1984; Akoun *et al.*, 1985; Esscher *et al.*, 1985; Cooper *et al.*, 1985) have demonstrated significantly higher levels of NSE in SCLC compared to other forms of lung cancer, with significant elevation of serum levels of NSE in patients with extensive compared to limited disease in SCLC. These observations were confirmed in our study, however, the median serum values of NSE in those patients with SCLC and extensive disease were higher than in limited disease but did not achieve statistical significance. Elevated serum levels of NSE ($\geq$12.5 ng ml$^{-1}$) were observed in 41% (13/32) in our patients with NSCLC compared to 18% (45/254) in previous studies (Ariyoshi *et al.*, 1983; Pahlman *et al.*, 1984; Akoun *et al.*, 1985; Cooper *et al.*, 1985; Esscher *et al.*, 1985). Interpretation of raised serum NSE levels in SCLC and NSCLC depend upon accurate histological classification, the neuroendocrine properties expressed by the tumour, and the upper limit of normal for NSE set for by the type of radioimmunoassay used. Further comparison of serum NSE values in NSCLC show that 77% were raised in the range 13–25 ng ml$^{-1}$ in our study and in 69% in previous studies, with 23% and 31% respectively >25 ng ml$^{-1}$. The use of a more discriminative upper limit for NSE of >25 ng ml$^{-1}$ will result in a decreased sensitivity and a higher specificity. Overall NSE as a tumour marker in lung cancer is potentially useful in SCLC in assessing disease extent, monitoring therapy and sub-clinical relapse, however, the pretreatment values were normal in 41% of our SCLC patients and in up to 30% in previous studies. Further clinical studies are required in larger numbers of patients with NSCLC with regard to neuroendocrine behaviour and subsequent tumour response to therapy.

The median values of serum PHI in both SCLC and

**Table IV** Alkaline phosphatase (AP), gamma-aminotranspeptidase (GGT), aspartate aminotransferase (AST), lactic dehydrogenase (LDH) and serum albumin and type and stage of lung tumour

| Patients | AP (IU l$^{-1}$) Median (range) | n | No. (%) $\geq$140 IU l$^{-1}$ | GGT (IU l$^{-1}$) Median (range) | n | No. (%) $\geq$50 IU l$^{-1}$ | AST (IU l$^{-1}$) Median (range) | n | No. (%) $\geq$40 IU l$^{-1}$ | LDH (IU l$^{-1}$) Median (range) | n | No. (%) $\geq$600 IU l$^{-1}$ | Albumin (g l$^{-1}$) Median (range) | n | No. (%) $\leq$35 g l$^{-1}$ |
|---|---|---|---|---|---|---|---|---|---|---|---|---|---|---|---|
| Total (*n*=73) | 138.8 (33–998) | 73 | 35 (48) | 83.3 (16–1,463) | 66 | 45 (69) | 28.3 (11–322) | 72 | 23 (32) | 468 (211–998) | 54 | 18 (33) | 37.4 (29–48) | 73 | 21 (29) |
| SCLC (*n*=41) | 104 (33–998) | 41 | 15 (37) | 58 (16–1,463) | 39 | 21 (54) | 27.5 (12–219) | 40 | 10 (25) | 479 (290–998) | 30 | 9 (30) | 40 (31–46) | 41 | 6 (15) |
| Limited (*n*=19) | 96 (33–181) | 19 | 3 (16) | 36 (16–130) | 19 | 8 (42) | 23.4 (13–65) | 19 | 2 (11) | 424 (290–998) | 15 | 5 (33) | 41.4 (34–46) | 19 | 2 (11) |
| Extensive (*n*=22) | 145.5[a] (61–998) | 22 | 12 (55) | 83.5[b] (19–1,463) | 20 | 13 (65) | 35[c] (12–219) | 21 | 8 (38) | 485 (293–998) | 15 | 4 (27) | 38 (31–45) | 22 | 4(18) |
| NSCLC (*n*=32) | 145.5 (59–750) | 32 | 18 (56) | 89 (29–552) | 27 | 21 (78) | 27.5 (11–130) | 32 | 11 (34) | 411 (211–998) | 24 | 8 (33) | 34.7[d] (29–48) | 32 | 15 (47) |
| Limited (*n*=6) | 141 (59–605) | 6 | 3 (50) | 287 (34–443) | 5 | 4 (80) | 28 (17–34) | 6 | 6 (100) | 432 (357–780) | 5 | 1 (20) | 38 (29–48) | 6 | 2 (33) |
| Extensive (*n*=26) | 145.5 (76–750) | 26 | 15 (58) | 87.5 (29–552) | 22 | 17 (77) | 26.5 (11–130) | 26 | 11 (42) | 409 (211–998) | 19 | 7 (37) | 34.5 (30–45) | 26 | 13 (50) |

[a]$P<0.01$; [b]$P<0.001$; [c]$P<0.05$; [d]$P<0.005$.

**Table V** The relationship of response to therapy and the number and percentage of normal and abnormal values for tumour markers, alkaline phosphatase, gamma GT and serum albumin in 69 treated patients with lung cancer

| | *NSE* $(ng\,ml^{-1})$ | | *PHI* $(IU\,l^{-1})$ | | *CC* $(mg\,l^{-1})$ | |
|---|---|---|---|---|---|---|
| *Response* | *Normal* | *≥12.5* | *Normal* | *≥120* | *Normal* | *≥55* |
| CR | 8 (57%) | 6 (43%) | 3 (21%) | 11 (79%) | 9 (64%) | 5 (36%) |
| PR | 13 (50%) | 13 (50%) | 5 (20%) | 20 (80%) | 15 (58%) | 11 (42%) |
| NR | 13 (45%) | 16 (55%) | 1 (3.5%) | 28 (96.5%) | 12 (41%) | 17 (59%) |
| | $\chi^2=0.58$, $P>0.7$ | | Fisher's exact test, $P>0.08$ | | $\chi^2=2.5$, $P>0.2$ | |
| | *AP* $(IU\,l^{-1})$ | | *GGT* $(IU\,l^{-1})$ | | *Albumin* $(g\,l^{-1})$ | |
| | *Normal* | *≥140* | *Normal* | *≥50* | *Normal* | *≤35* |
| CR | 11 (79%) | 3 (21%) | 7 (54%) | 6 (46%) | 13 (93%) | 1 (7%) |
| PR | 17 (65%) | 9 (35%) | 14 (56%) | 11 (44%) | 22 (85%) | 4 (15%) |
| NR | 10 (35%) | 19 (65%) | 5 (18%) | 22 (82%) | 17 (59%) | 12 (41%) |
| | $\chi^2=9.211$, $P<0.01$ | | $\chi^2=8.896$, $P<0.02$ | | Fisher's exact test, $P<0.03$ | |

**Table VI** The number of abnormal haematological and biochemical indices and response to therapy

| | *Number of abnormal haematological and biochemical indices* | |
|---|---|---|
| *Response* | *1 to 6* | *≥7* |
| CR | 12 (86%) | 2 (14%) |
| PR | 16 (62%) | 10 (38%) |
| NR | 10 (34%) | 19 (66%) |

$\chi^2=10.72$, $P<0.005$.

NSCLC were increased at 156.6 IU $l^{-1}$ and 173.5 IU $l^{-1}$ respectively, but measurement of the glycolytic enzyme did not differentiate between the type or stage of lung cancer. In an earlier report (West *et al.*, 1962) 72% of 126 patients with lung cancer had elevated serum levels of PHI with higher values occurring in those with distant metastasis. Bodansky (1954) had previously observed a close correlation of serum PHI and the presence of bone and hepatic metastases in carcinoma breast. Conflicting views (Schwartz *et al.*, 1985; d'Eril *et al.*, 1986) have been expressed on the specificity and sensitivity of PHI in lung cancer. Schwartz *et al.* (1985) stated that in 77 patients with lung cancer PHI exhibited a high sensitivity in detecting lung cancer with good specificity for normal subjects and in patients with benign lung tumours, but was less specific in benign respiratory diseases. Forty-four patients with SCLC had the highest values for PHI and the levels in those patients with localised disease were not significantly different from patients with advanced disease, and suggested that PHI may be a useful marker in the early detection of lung cancer. In contrast, d'Eril *et al.* (1986) stated that PHI in lung cancer had only fair sensitivity and a very low specificity, in that the mean values of PHI were similar to other neoplasms and other non-neoplastic respiratory diseases, and there was no significant difference in the mean values between early and metastatic lung cancer. Our study similarly showed that PHI could not differentiate the type of lung tumour or provide additional information for staging purposes.

In the present study, serum levels for CC were significantly higher in NSCLC, compared to SCLC. Gropp *et al.* (1980) reported that the incidence of CC using the % inhibition of C1q binding uptake was similar in the different histological types of lung cancer in 100 patients. Their study also demonstrated higher levels of CC in 75% of patients with metastases in contrast to only 25% in those with localised disease. Similar observations have been made in acute leukaemia, lymphoma and other solid tumours (Heimer & Klevi, 1976; Theofilopoulus *et al.*, 1976; Teshima

**Table VII** Pre-treatment variables

| *Group A (P>0.05)* | *Group B (0.01>P<0.05)* | | *Group C (P<0.01)* | |
|---|---|---|---|---|
| Age | Lung pathology | (0.0403) | LDH | (0.0067) |
| Sex | Lung metastasis | (0.0341) | Albumin | (0.0017) |
| White cell count | Haemoglobin | (0.0351) | Stage | (0.0036) |
| Platelets | Urea | (0.0467) | [a]**Liver metastasis** | **(0.00002)** |
| Sodium | Alkaline phosphatase | (0.0168) | [a]**4 Abnormal indices** | **(0.000001)** |
| Potassium | Asp aminotransferase | (0.0422) | | |
| Chloride | Gamma GT | (0.0482) | | |
| Calcium | | | | |
| Bicarbonate | | | | |
| Immune complexes | | | | |
| NSE | | | | |
| PHI | | | | |
| Site of tumour in lung | | | | |
| Interval first symptom to diagnosis | | | | |
| Interval diagnosis to treatment | | | | |
| Mediastinal nodes | | | | |
| Other metastasis | | | | |
| Bone metastasis | | | | |
| Lymph nodes | | | | |
| Soft tissue metastasis | | | | |

[a]Significant variables in final model: **bold letters**.
*Note*: (i) ALT, ESR, bone marrow metastasis were not included as their numbers were too few. (ii) Serum protein excluded as all measurements were within the normal range.

**Figure 4** Survival curves with abnormal haematology and biochemical indices and the presence of hepatic metastases in 73 patients with lung cancer.

*et al.*, 1977). In contrast, we have not shown significantly higher levels of CC in patients with lung cancer and extensive disease. The assay for CC employed in this study has been previously shown (Levinson *et al.*, 1984) to correlate highly with C1q binding technique, and therefore technique seems unlikely to explain the differing results.

It was evident that in the 41 patients with SCLC, the median values of AP, GGT and AST were significantly elevated in those patients with extensive disease compared to those with limited disease. The levels of GGT, alkaline phosphatase and oxidative liver enzymes have been observed previously to rise at a relatively late stage of tumour dissemination (West *et al.*, 1962; Neville & Cooper, 1976).

The levels of NSE, PHI and CC did not correlate with the bulk of disease (i.e. the number of metastatic sites involved). The use of other tumour markers (ACTH, calcitonin and CEA) may allow a better correlation with the extent of disease (Havemann *et al.*, 1985). A recent study by Cooper *et al.* (1987) in SCLC showed that NSE was a more sensitive indicator of disease activity and monitoring therapy compared to CEA and acute phase proteins.

The pre-treatment serum values of the tumour markers NSE, PHI and CC did not predict the patients' response to therapy and were not significant prognostic factors. A greater proportion of patients with an elevated AP or GGT were non-responders, and a greater proportion of patients who went into complete remission or partial response had serum levels of AP or GGT within their normal range or a serum albumin $>35\,g\,l^{-1}$. Other individual haematology or biochemical indices at presentation were not predictive factors for response to therapy in the 69 evaluable patients with lung cancer. However, the presence of more than 7 abnormal indices was significantly associated with a greater proportion of non-responders, and conversely a greater proportion were complete responders and partial responders when the number of abnormal indices was less than 7.

Only two out of 32 pre-treatment variables were significantly related to prognosis after evaluation in a multiple regression analysis, although other variables, e.g., tumour stage, serum albumin, alkaline phosphatase, etc., were significant on univariant analyses, but the presence of more than four abnormal indices and liver metastasis were the only important, independent variables. Once they had been included in the model no other variable contained any significant additional prognostic information.

In our study, the presence of 4 abnormal indices was the most important prognostic factor after evaluation in a multiple regression analysis and it was not possible to identify which combination of individual haematological and biochemical variables specifically affected survival due to the small numbers involved. However, as in previous studies (Cohen *et al.*, 1981; Souhami *et al.*, 1985; Cerny *et al.*, 1987) the use of simple laboratory parameters provide as much information for prognosis as the other staging procedures of scans, bone marrow, etc. Cohen *et al.* (1981) identified a high plasma albumin and haemoglobin as the most influential prognostic factors in 56 patients with SCLC. Souhami *et al.* (1985) analysed 371 patients with SCLC and reported Karnofsky performance score, serum albumin, sodium, alkaline phosphatase and disease extent as independently significantly related to survival. Similarly, our own group (Cerny *et al.*, 1987) found lactic dehydrogenase, sodium, Karnofsky performance score, alkaline phosphatase, tumour stage and bicarbonate, as important prognostic factors in 407 patients with SCLC. The presence of liver metastasis was the next most important independent pre-treatment variable in our present study ($P<0.008$). Ihde *et al.* (1981) stated that the median survival progressively worsened with increasing number of metastatic sites, and that metastasis to liver and brain significantly shortened survival in 106 patients with SCLC treated with chemotherapy. Similar observations on the presence of hepatic metastasis on survival have been made in both treated and untreated lung cancer patients (Zelen, 1973; Lanzotti *et al.*, 1977).

Earlier studies using univariate analysis reported performance score and extent of disease as the most important prognostic factors in lung cancer (for review, Stanley, 1985), but included only a few of the plethora of patient factors which may provide pertinent information in the assessment of survival. More recent studies (Cohen *et al.*, 1981; Souhami *et al.*, 1985; Cerny *et al.*, 1987) have shown that the inclusion of standard haematological and biochemical indices and clinical features are required in a multiple regression analysis for predicting survival in lung cancer. The present study has shown that NSE, PHI and CC as pre-treatment biological markers did not provide any additional information to existing staging investigations nor predict the response to therapy or the outcome in this group of 73 patients with lung cancer. The number of abnormal routine laboratory parameters was the most important prognostic factor.

We would like to thank Miss Gail Smart for her help in preparing and typing the manuscript.

### References

AKOUN, G.M., SCARNA, H.M., MILLERON, B.J., BENICHOU, M.P. & HERMON, D.P. (1985). A marker for disease extent and response to therapy for small cell lung cancer. *Chest*, **87**, 39.

ARIYOSHI, Y., KATO, K., ISHIGURO, Y., OTA, K., SATO, T. & SUCHI, T. (1983). Evaluation of serum neuron specific enolase as a tumour marker for carcinoma lung. *Gann.*, **74**, 219.

BAYLIN, S.B., GOODMAN, G. & SHAPER, J.H. (1982). Analysis of cell surface proteins as a means to study neuroendocrine differentiation in the spectrum of human lung cancers. In *Systemic Role of Regulatory Peptides*, Bloom *et al.* (eds) p. 307. Verlag: Stuttgart.

BODANSKY, O. (1954). Serum phosphohexose isomerase in cancer. *Cancer*, **7**, 1200.

CARNEY, D.N., IHDE, D.C. & COHEN, M.H. & 4 others (1982). Serum neuron-specific enolase: A marker for disease extent and response to therapy of small cell lung cancer. *Lancet*, **i**, 583.

CARNEY, D.N. (1987). Clinical relevance of lung cancer biology. *Br. J. Cancer*, **56**, 884.

CERNY, T., BLAIR, V., ANDERSON, H., BRAMWELL, V. & THATCHER, N. (1987). Pre-treatment prognostic factors and scoring systems in 407 small cell lung cancer patients. *Int. J. Cancer*, **39**, 146.

COHEN, M.H., MAKUCH, R., JOHNSTON-EARLY, A. & 4 others (1981). Laboratory parameters or an alternative to performance status in prognostic stratification of patients with small cell lung cancer. *Cancer Treatment Rep.*, **65**, 187.

COOPER, E.H., MUER, M.F., PEAKE, M.D., JORGENSON, L. & HANSEN, H.H. (1987). Neuron-specific enolase, CEA and acute phase proteins in the monitoring of small cell lung cancer. *Br. J. Cancer*, **56**, 890.

COX, D.R. (1972). Regression models and life tables. *J. Statis. Soc.* (A), **35**, 185.

D'ERIL, G.M., PAVESI, F., LOTZNIKER, M. & MORATTI, R. (1986). More on phosphohexose isomerase as a tumour marker. *Clin. Chem.*, **32**, 1242.

DHILLON, A.P., RODE, J., DHILLON, D.P., MOSS, E., ON, R.J. & SPIRO, S.G. (1985). Neural markers in carcinoma of the lung. *Br. J. Cancer*, **51**, 645.

ESSCHER, T., STEINHOLTZ, L., BERGH, J., NOU, E., NILSSON, K. & PAHLMAN, S. (1985). Neuron specific enolase: A useful diagnostic serum marker for small cell carcinoma of the lung. *Thorax*, **40**, 85.

GAZDAR, A.F., CARNEY, D.N. & MINNA, J.D. (1983). The biology of non-small cell lung cancer. *Semin. Oncol.*, **10**, 3.

GROPP, C., HAVEMANN, K., SCHERFE, T. & AX, W. (1980). Incidence of circulating immune complex in patients with lung cancer and their effect on antibody dependent cytotoxicity. *Oncology*, **37**, 71.

HAVEMANN, K., HOLLE, R. & GROPP, C. (1985). Prospective multicentre study of hormone markers in small cell lung cancer. In *Peptide Hormones in Lung Cancer*, Havemann *et al.* (eds). Springer-Verlag: Berlin.

HEIMER, R. & KLEVI, G. (1976). Circulating immune complexes in sera of patients with Burkitt's lymphoma and nasopharyngeal carcinoma. *Int. J. Cancer*, **18**, 310.

IHDE, D.C., MAKUCH, R.W., CARNEY, D.N. & 4 others (1981). Prognostic implications of stage of disease and sites of metastases in patients with small cell carcinoma of the lung treated with intensive combination chemotherapy. *Am. Rev. Resp. Dis.*, **123**, 500.

JOHNSON, D.H., MARANGOS, P.J., FORBES, J.T. & 4 others (1984). Potential utility of serum neuron specific enolase levels in small cell carcinoma of the lung. *Cancer Res.*, **44**, 5409.

KAPLAN, E.L. & MEIER, P. (1958). Non-parametric estimation from incomplete observations. *J. Am. Statist. Assoc.*, **53**, 457.

KATO, K., ASAI, R., SHIMIZU, A., SUZUKI, F. & ARIYOSHI, Y. (1983). Immunoassay of three enolase isoenzymes in human serum and in blood cells. *Clin. Chem. Acta.*, **127**, 353.

KRAPF, F., RENGER, B., SCHEDEL, I., LEINDECKER, K., LEYSSENS, H. & DEIEHER, H. (1982). PEG-precipitation laser nephelometer technique for detection and characteristics of circulating immune complexes in human sera. *J. Immunol. Meth.*, **54**, 107.

LANZOTTI, V.L., THOMAS, D.R., BOYLE, L.E., SMITH, T.L., GEHAN, E.A. & SAMUELS, M.L. (1977). Survival with inoperable lung cancer. An integration of prognostic variables based on simple clinical criteria. *Cancer*, **39**, 303.

LEVINSON, S.S., GOLDMAN, J.O. & FELDKAMP, C.S. (1984). Anti-IgG binding test to assay circulating IgG-containing immune complexes from polyethylene glycol precipitates. *Clin. Chem.*, **90**, 1502.

McDOWELL, E.M. & TRUMP, B.F. (1981). Pulmonary small cell carcinoma showing tripartate differentiation in individual cells. *Hum. Pathol.*, **12**, 286.

NEVILLE, A.M. & COOPER, E.H. (1976). Biochemical monitoring of cancer. A review. *Ann. Clin. Biochem.*, **13**, 283.

PAHLMAN, S., ESSCHER, T., BERGH, J., STINHOLTZ, L., NOU, E. & NILLSON, K. (1984). Neuron specific enolase as a marker for neuroblastoma and small cell carcinoma of the lung. *Tumour Biology*, **5**, 119.

PETO, R., PIKE, M.C., ARMITAGE, P. & 7 others (1977). Design and analysis of randomised clinical trials requiring prolonged observation of each patient. II. Analysis and examples. *Br. J. Cancer*, **35**, 1.

ROWAN, R.M. (1978). The assay of phosphoglucose isomerase in human serum. *Medical Lab. Sci.*, **35**, 155.

SCHWARTZ, M.K., DNISTRIAN, A.M., STANKIEVIC, R., MINDICINO, H. & SCHWARTZ, D. (1985). Phosphohexose isomerase (PHI) as a marker in lung cancer. *Clin. Chem.*, **31**, 983.

SIDHU, G. S. (1980). The ultrastructure of malignant epithelial neoplasms of the lung. *Path. Ann.*, **1**, 235.

SOUHAMI, R.L., BRADBURY, I., GEDDES, D.M., SPIRO, S.G., HARPER, P.G. & TOBIAS, J.S. (1985). Prognostic significance of laboratory parameters measured at diagnosis in small cell carcinoma of the lung. *Cancer Res.*, **45**, 2878.

STANLEY, K.E. (1985). Prognostic factors in lung cancer. In *Lung Cancer*, Aisner, J. (ed), Churchill Livingstone. *Contemp. Issues Clin. Oncol.*, **3**, 41.

TAPIA, F.J., BARBOSA, A.J.A., MARANGOS, P.J. & 3 others (1981). Neuron specific enolase is produced by neuroendocrine tumours. *Lancet*, **i**, 808.

TESHIMA, H., WANEBO, H., PINSKY, C. & DAY, N.K. (1977). Circulating immune complexes detected by $^{125}$I-C1q deviation test in sera of cancer patients. *J. Clin. Invest.*, **59**, 1134.

THEOFILOPOULUS, N., WILSON, C.B. & MULLER-EKERHARD, H.J. (1976). C1q deviation test for the detection of immune complexes, aggregates of IgG, and bacterial products in human serum. *J. Exp. Med.*, **142**, 139.

WEST, M., SCHWARTZ, M.A., WALSH, W.S. & ZIMMERMAN, H.J. (1962). Serum enzymes in disease. Glycolytic and oxidative enzymes and transaminases in patients with cancer of the lung. *Cancer*, **15**, 931.

WILLIS, R.A. (1948). *Pathology of Tumours*. Butterworth, London.

ZELEN, M. (1973). Keynote address on biostatistics. *Cancer Chemotherap. Rep.*, **4**, 31.